# A Comparison Study of Posture and Fatigue of Neck According to Monitor Types (Moving and Fixed Monitor) by Using Flexion Relaxation Phenomenon (FRP) and Craniovertebral Angle (CVA)

**DOI:** 10.3390/ijerph17176345

**Published:** 2020-08-31

**Authors:** Kyeong-Hee Choi, Min-Uk Cho, Chae-Won Park, Seoung-Yeon Kim, Min-Jung Kim, Boram Hong, Yong-Ku Kong

**Affiliations:** 1 Department of Industrial Engineering, Sungkyunkwan University, Suwon 16419, Korea; kyunghe7@naver.com (K.-H.C.); crayonmm@naver.com (M.-U.C.); cwrachel@naver.com (C.-W.P.); kimsy9035@naver.com (S.-Y.K.); xlsk1013@naver.com (M.-J.K.); 2 DOTHEAL Co., Ltd., Suwon 16690, Korea; brian@dotheal.com

**Keywords:** forward head posture (FHP), flexion relaxation phenomenon (FRP), visual display terminal (VDT), neck fatigue, craniovertebral angle (CVA)

## Abstract

This study quantified the neck posture and fatigue using the flexion relaxation phenomenon (FRP) and craniovertebral angle (CVA); further, it compared the difference between the level of fatigue and neck posture induced by two types of monitors (regular fixed monitor and moving monitor). Twenty-three male participants were classified into two groups—the low-flexion relaxation ratio (FRR) group and the normal-FRR group, depending on the FRR value. All participants performed a document task for 50 min using both types of monitors. It was found that the FRR values significantly decreased after the documentation task. The CVA analysis showed that the moving monitor’s frequency of forward head posture (FHP) was lower than that for the fixed monitor. Overall, the moving monitor worked better than the fixed monitor; this can be interpreted as proof that such monitors can reduce neck fatigue.

## 1. Introduction

Advances in technology have increased the use of visual display terminals (VDTs) in industrial fields; especially, the wide use of personal computers has increased dramatically. Along with the growing use of VDT, work-related musculoskeletal disorders (WMSDs) of VDT workers have emerged as a serious problem in recent years [1].

During their work hours, most VDT workers watch the monitors for a long time; this may lead to awkward postures of the neck and head. Especially, the forward head posture (FHP) increases the loads on the necks and shoulders of people who spend many hours working on their computers; it causes back and neck pain in 30% of VDT workers [2]. FHP has been defined as a posture in which the head is in front of the body-center; this leads to a 3.6 times larger load than that during a normal posture [3]. Thus, FHP has been reported as the leading cause of WMSDs, such as pains of neck, shoulder, and headaches [4,5,6]. To diagnose FHP, the craniovertebral angle (CVA) has frequently been evaluated in many related studies [7,8,9]. CVA is the angle between a horizontal line passing through the spinous process of C7 and a line extending from C7 to the tragus of the ear [10]; a lower CVA (average CVAs with pain and without pain groups are 44.44 and 48.63, respectively) indicates higher FHP [6,11].

During computer work, the cervical erector spinae (CES) muscle is important for efficient activation and support for the task. Yoo et al. (2011) reported that the fatigue of CES muscle owing to the VDT task could be quantified with the flexion relaxation phenomenon (FRP), which is defined as an electrical silence response in the erector spinae (ES) muscles during the full flexion of the trunk [2,12]. It is caused by a shift in load-sharing from the active structures (ES muscles) to passive structures of the spine (ligament, capsules, and vertebral disk) [13,14].

The FRP of the cervical spine is similar to those observed for the ES muscle. In the cervical spine, during neck flexion, the cervical extensors (active structures) gradually increase their muscle activation to compensate for the increase in the head’s load [15]. When the head is fully flexed, the load on the muscles (active structures) is transferred to the passive structures, resulting in a reduction, or silence, of the myoelectric activity of muscles [16]. This coordination between two subsystems (active and passive) is important to ensure the mechanical stability of the spine and the neural subsystem [17].

The FRP parameters, such as flexion relaxation ratio (FRR), have been widely used. FRR is defined as the ratio of the maximum activation in the re-extension phase to the average activation in the full-flexion phase (silence period) [15]. The FRR is a very useful indicator to distinguish between healthy subjects and those with neck pain [18] because patients experiencing neck pain have been reported to not show the FRP due to abnormal recruitment patterns. Many researchers have reported a significant difference in the FRR value between healthy and neck-pain patients, and a relatively lower value of FRR has been found in neck-pain patients [18,19].

Besides, many researchers have reported that fatigue influences the stability of the cervical spine, resulting in a decrease in the FRR value [20]. Even a short period of fatigue could affect the cervical spine stability by transferring loads to the passive tissues and substantially increasing muscle activity [21], so the FRR is a sensitive index of fatigue.

Electromyography (EMG) frequency is also a widely used quantification index to describe muscle fatigue, and there is a shift in the EMG power spectrum toward low frequencies when muscular fatigue occurs [22]. However, Murata et al. (2003) reported that it was difficult to evaluate the local muscular fatigue using EMG during the VDT task because such tasks involve many movements that require the activation of different muscles and motor units. In addition, an EMG recording during the VDT task is not recommended because EMG signals are subject to interference when movement occurs [23]. Niu et al. (2008) also reported that there was no significant difference in the mean power frequency because of muscle fatigue during the VDT task [24].

Musculoskeletal disorders owing to work involving a VDT could be prevented by maintaining an appropriate neck posture and regular stretching [25]; thus, many guidelines for the correct working posture and tools for posture correction have been developed for VDT workers. However, these methods have been found to be ineffective for posture correction because users should perform them consciously, but this is inconvenient in the office environment. Further, there are very few studies about the tools designed to correct the neck posture for VDT workers.

To compensate for these limitations, a monitor that could actively produce adjustments in a user’s neck posture has been developed. FHP is frequently observed when the height of the monitor is lower than the eye position [26]. Because the monitor’s location affects the user’s neck posture, the solution is to periodically change the height, angle, and horizontal distance of the monitor. In addition, the moving monitor is designed so that its movement is too slow to be perceptible to the users; this is because the monitor’s movement could be distracting if the user notices it. The noise from the monitor’s motors that may affect the user’s attention is also minimized, thereby making it difficult for the users to recognize it.

The objectives of this study were as follows: (1) quantify the neck posture and fatigue of neck muscle, the cervical erector spinae (CES), using CVA and FRR, respectively, and (2) compare the difference in the level of fatigue and neck posture induced by the two types of the monitor (regular fixed monitor and moving monitor) for males during 50 min of documentation writing task.

Based on existing research results, the first hypothesis was that there would be significant differences in FRR, CVA, and CVA ratio associated with neck fatigue depending on neck health conditions. The second hypothesis was that there would be significant differences in FRR, CVA, and CVA ratio depending on the type of monitor. Finally, the third hypothesis was that there would be significant differences in FRRs before and after documentation writing task.

## 2. Materials and Methods

### 2.1. Participants

A total of 23 males were recruited through advertisements within the graduate student population who worked on computers for more than four hours a day. Exclusion criteria were a history of pains or musculoskeletal disorders in their neck, shoulder, upper limbs, and back. All participants were informed about the content and purpose of this study and provided a consent form before the start of the study. All participants were also informed about the benefits and risks of participation and could withdraw from the study at any time. There is no any ethics issues in this study. Each participant was paid at a rate of $15.00 per hour for participation. To identify the effect of neck health conditions, participants were divided into two groups (low-FRR and normal-FRR) based on the threshold FRR value of 2.5, which was used to identify the presence or absence of FRP [15,27]. The participants with FRR value higher than 2.5 (16 males) were placed in the normal-FRR group, and the remaining (seven males) were classified as the low-FRR group. Anthropometric data of the participants, such as age and weight, were collected to establish the experimental setting (Table 1).

### 2.2. Experimental Design

In this study, the following three variables were the independent variables: (1) neck health condition (low-FRR and normal-FRR group)—Hypothesis 1; (2) monitor type (moving and fixed)—Hypothesis 2; and (3) before and after the task (pre and post)—Hypothesis 3.

**Figure 1:**
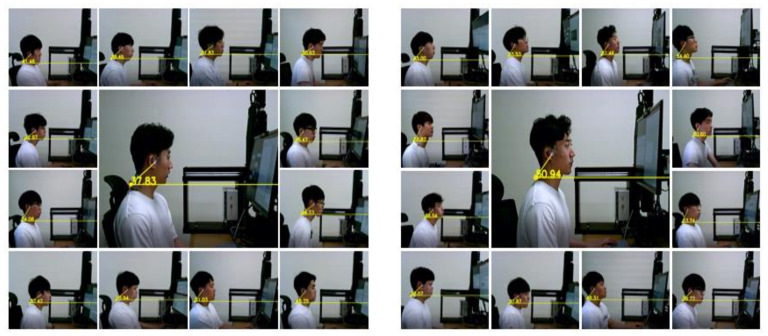
Fixed monitor test (**left**) and moving monitor test (**right**).

For the monitor types, the moving monitor can change—at an imperceptible speed—the height and degree to induce a change in the user’s neck postures, whereas the fixed monitor is a regular monitor without any movement. The custom-made monitor system that can move in all directions (the movements were up/down, left/right, and tilting forward/backward) was developed for this study (Figure 1, right). This system also allowed adjusting the speed of movement and the moving trajectories. If a movement mechanism was applied to the monitor, it was defined as a “moving monitor”. Otherwise, it was defined as a “fixed monitor”, similar to those used in offices (Figure 1, left). The speed of moving and tilting was set to 0.6 mm/s and 0.05 mm/s, respectively; this was done to prevent the participants from being aware of the monitor’s movements. A cycle of movement was set as 10 min.

Five levels of dependent variables were selected to quantify the neck fatigue and posture: (1) FRR; (2) CVA; (3) ratio of the CVA. The definitions of the dependent variables are as follows.

**Figure 2:**
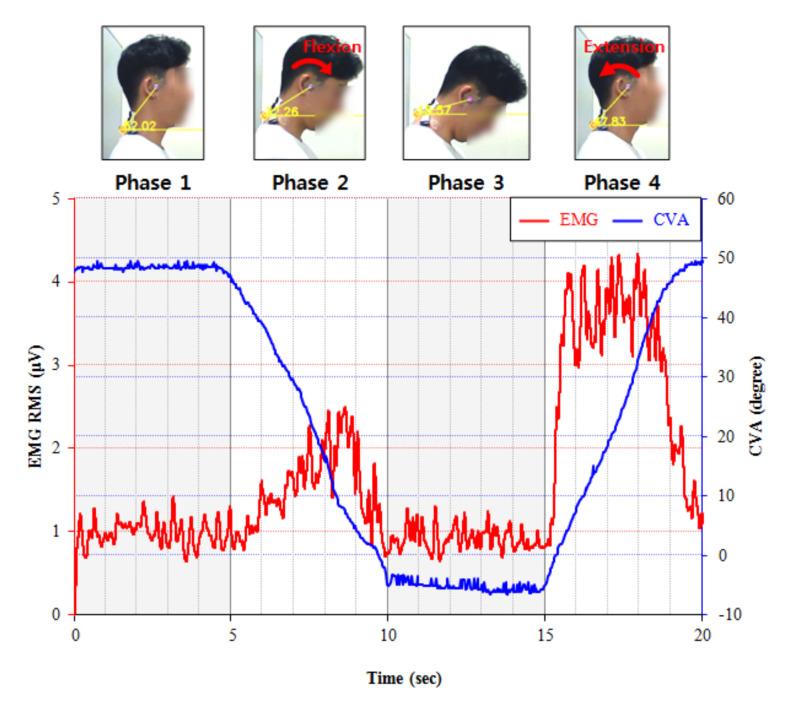
CVA (craniovertebral angle) and EMG (electromyography) data during the cervical flexion relaxation trials.

1. FRR: The FRR has been widely used to quantify the relaxation ability of the extensor muscles of the neck. The FRR, as calculated by using Equation (1), is defined as the ratio of the maximum EMG value in the extension phase (phase 4) to the average EMG value in the full flexion phase (phase 3), as shown in Figure 2 [15].

(1) $$FRR=\frac{Maximum\;EMG\;in\;Phase\;4}{Average\;EMG\;in\;Phase\;3}$$

The wireless EMG system (TeleMyo 2400 DTS, Noraxon, Arizona, USA) was used to measure the muscle activity of CES muscles during the flexion relaxation (FR) test and main study. The raw EMG data were digitally filtered with a bandwidth of 80–250 Hz and sampled at 1000 Hz. Unfortunately, neck muscle (CES) EMG recordings are often contaminated by the electrocardiogram (ECG) and may result in misinterpretations. Thus, an electrocardiogram (ECG) reduction filter was also applied to minimize the effect of the ECG.

**Figure 3:**
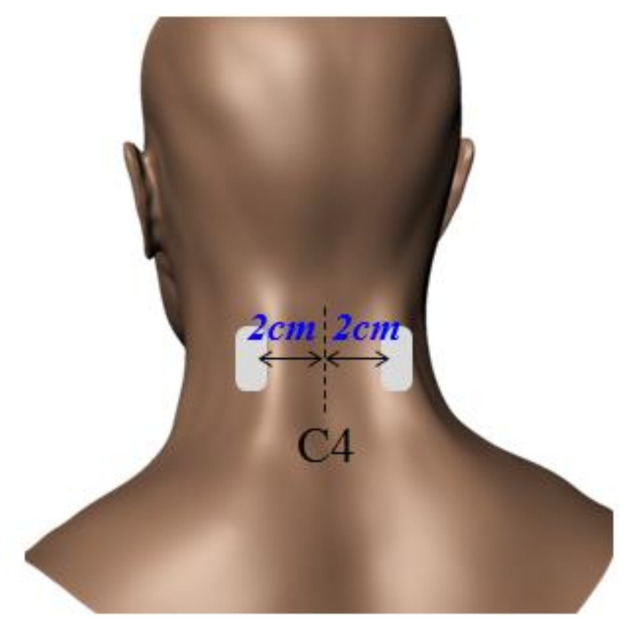
Placements of electrodes.

After skin preparation with alcohol, the surface Ag-AgCl electrodes were secured with double-sided tape over the right and left sides of the CES muscle; 2 cm lateral to the C4 spinous process; parallel alignment with the direction of the muscle fibers, following the placement recommendations of Murphy and Marshall (2010), as shown in Figure 3 [18]. The electrodes’ positions were marked on the skin with a surgical marker to maintain the same experimental conditions for both moving and fixed monitors.

The reason for selecting the CES muscle in this study was that, as stated in the introduction, CES muscle is essential for efficient activation and support for the task. The fatigue of CES muscle owing to the VDT task could be quantified with the flexion relaxation phenomenon (FRP), which is defined as an electrical silence response in the erector spinae (ES) muscles during the full flexion of the trunk [2,12].

**Figure 4:**
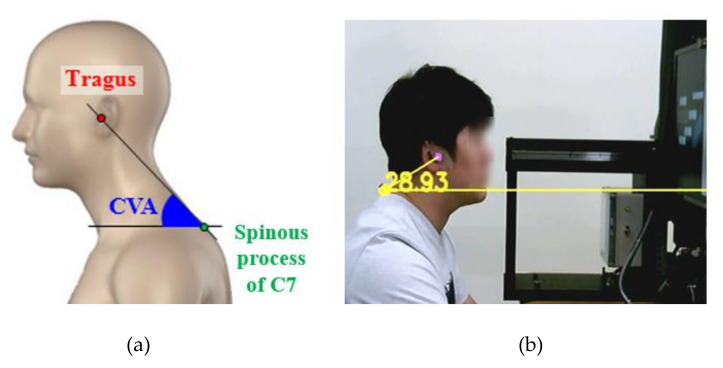
(**a**) Craniovertebral angle (CVA); (**b**) Open source computer vision (OpenCV).

2. CVA (degree): The CVA is the angle between a horizontal line passing through the spinous process of C7, and a line extending from the tragus of the ear to C7 (Figure 4a) was also observed to assess the angles of the neck postures in this study. A webcam (HD WebCam C270, Logitech, Seoul, Korea) was installed on the right side of the participants to measure the CVA. The CVA detection program was developed based on the OpenCV-Python; it could detect two markers attached to the participant’s Tragus and C7 and calculate the CVA in real-time (Figure 4b).

3. The ratio of the CVA: Based on the FHP, the CVA ratio was considered as reflecting good, fair, and bad posture. If the CVA was higher than 48.7°, it was defined as a “good” posture. A CVA range of 43.8–48.7° and a value lower than 43.8° were defined as “fair” and “bad” postures, respectively [6].

### 2.3. Experimental Procedure

Before the experiment, all participants were informed about the purposes and procedures of this study; further, anthropometric data, such as height, weight, and age, were collected. Two markers were attached to the participant’s tragus and C7 to measure the CVA. Then, the electrodes were attached on both sides of the CES muscle.

Before and after the 50-min document writing task, the flexion relaxation (FR) test was conducted five times. All participants performed a practice round of the FR test to get accustomed to the process used and posture adopted in the FR test. Three minutes of rest time was provided to all participants to avoid fatigue. All participants were asked to maintain an upright trunk position and knees at 90° throughout all tasks.

**Figure 5:**
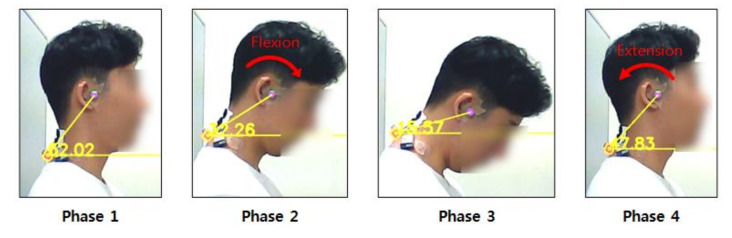
The procedure of flexion relaxation (FR) test.

The cervical FR test consisted of four phases: (1) neutral head position (phase 1); (2) cervical flexion (phase 2); (3) maintaining maximal cervical flexion (phase 3); (4) cervical extension (phase 4). In phase 1, participants were asked to maintain the neutral head posture and keep their eyes on a forward marker. They were then asked to lower their head slowly until the chin touched their upper chest (manubrium) (phase 2, cervical flexion). In phase 3, participants were asked to maintain the maximal cervical flexion posture for 5 s and then return to the neutral posture slowly in phase 4. Each phase lasted for 5 s, and a metronome was applied to standardize the speed and duration of all phases (Figure 5).

A document writing task with the moving and fixed monitors was performed on different days to minimize the effect of fatigue. The chair’s height was adjusted to align the participant’s eye level to the center of the screen. Microsoft office specialist (MOS) was selected as a document task, and all participants carried out the same task for 50 min. In this experiment, the sitting posture was not strictly controlled because the purpose of moving monitor is to naturally induce changes in the participant’s posture according to the monitor’s movement. Therefore, all participants were asked to conduct the document task while assuming a comfortable posture that they usually adopted.

### 2.4. Data Analysis

All statistical analyses of the measured values were performed using SPSS 18.0 statistical software (Lead Technologies, Inc., Chicago, IL, USA). Two-way analysis of variance (ANOVA) was used to compare the effect of neck health condition (low-FRR/normal-FRR) and monitor type (moving/fixed) on the CVA, the ratio of CVA, and FRR adopted. ANOVA was used to compare the effect of before/after the task (pre/post) on the FRR. When significant differences between groups were found by ANOVA, this was followed by post hoc testing with Tukey’s studentized range (HDS: honestly significant difference). The statistical significance level for all tests was set at *p* < 0.05.

## 3. Results

### 3.1. Craniovertebral Angle (CVA)

Although the results showed that there was no statistically significant difference in CVA according to the neck health condition (*p* = 0.079) and monitor type (*p* = 0.629), the low-FRR group (FRR < 2.5) had a lower CVA (41.7°) than the normal-FRR group (FRR ≥ 2.5)’s value of 45.6°. When working on the fixed monitor (43.1°), the CVA was also slightly lower than that when the moving monitor was used (44.2°).

### 3.2. The Ratio of Craniovertebral Angle (CVA)

The CVA was measured in real-time to examine the change of neck posture during the 50 min of the document writing task. According to the criteria for distinguishing FHP, the neck posture was classified into good, fair, and bad. If the CVA was higher than 48.7°, it was defined as a “good” posture. A CVA between 48.7° and 43.8° was defined as a “fair” posture, and a CVA lower than 43.8° was considered “bad” [6].

**Figure 6:**
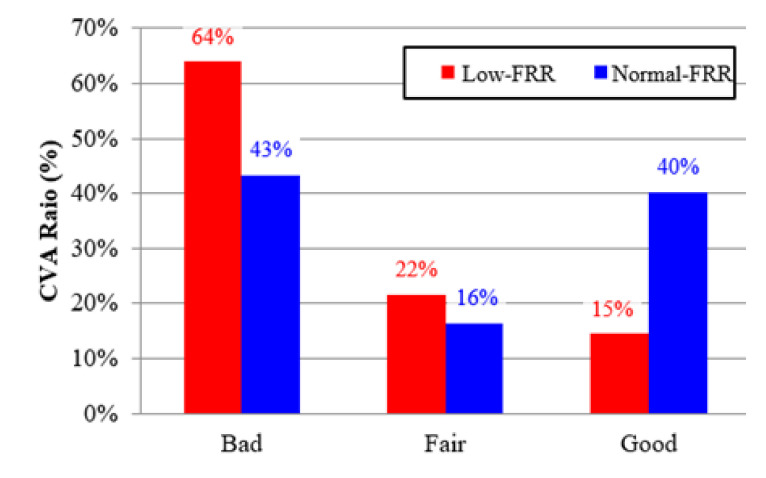
The effect of neck health on the CVA ratio.

The effect of the neck health condition on the ratio of CVA was statistically significant in this study (*p* = 0.001). In the low-FRR group, the CVA ratios of good, fair, and bad postures were 15%, 22%, and 64%, respectively; this means that the bad neck posture occurred more frequently than the other postures. In the normal-FRR group, the participants with fair and good postures accounted for 16% and 40%, respectively. The ratio of bad posture in the normal-FRR group was 43% in this study. It was noted that the normal-FRR group’s CVAs were more evenly distributed than those of the low-FRR group (Figure 6).

**Figure 7:**
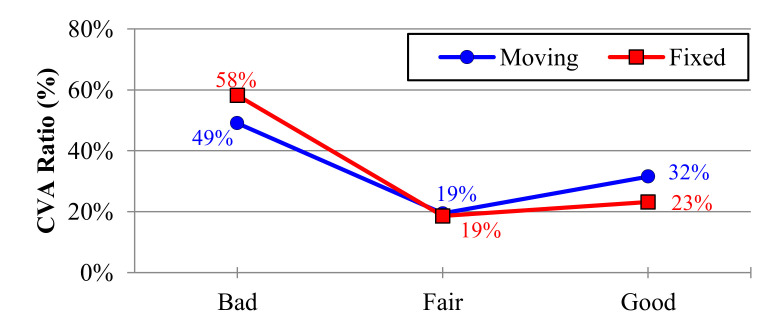
The main effect of the monitor type on the CVA ratio.

Although the CVA ratio was not statistically significant according to the monitor type or the interaction effect of monitor type and neck health condition, the percentage of good posture observations for the moving monitor (32%) was relatively higher than that for the fixed monitor (23%); while the ratio of bad posture for the moving monitor (49%) was relatively lower than that for the fixed monitor (58%), as shown in Figure 7.

In the normal-FRR group, the difference in the CVA ratio between the two types of monitors did not exceed 5%; however, the low-FRR group showed a higher difference according to the monitor type 14.6% more than the normal-FRR group. The bad posture ratio for the fixed and moving monitors was 71.2% and 56.6%, respectively (see Table 2), in the low-FRR group.

### 3.3. Flexion Relaxation Ratio (FRR)

The FRR value had a statistically significant difference between pre and post (*p* = 0.002). As expected, the FRR value decreased after the document writing task. The FRR value of post (2.79) was significantly lower than that of pre (3.11).

**Figure 8:**
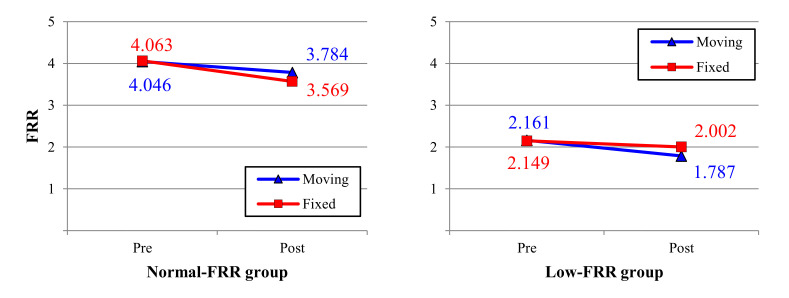
The interaction effect of monitor type and pre/post on neck health.

Although the interaction effect of neck health condition and monitor type on the FRR was not statistically significant, an interesting trend was found in the neck health condition. In the normal-FRR group, the FRR decrease in the fixed monitor (0.494) after the task was slightly higher than that for the moving monitor (0.262); in contrast to the normal-FRR group, the FRR decrease for the moving monitor (0.374) was higher than that for the fixed monitor (0.147) in the low-FRR group (Figure 8).

## 4. Discussion

This study was designed to evaluate the effect of a moving monitor on the improvement in the FHP. The participants were classified into two groups (normal-FRR and low-FRR) according to the FRR value to examine the difference in neck fatigue between the two groups.

### 4.1. Craniovertebral Angle (CVA)

FHP is a frequent occurrence during a prolonged VDT task that increases the fatigue of muscles supporting the head and neck [8,28]. FHP also increases loads on the joints and muscles of the neck and is related to chronic musculoskeletal disorders, fatigue, and pain [29,30]. Thus, it is very important to prevent this awkward posture in a workspace with VDTs. FHP can be described as an excessive anterior positioning of the head relative to a vertical reference line. FHP is usually quantified by the CVA, and lower CVA values indicate an unsuitable FHP. If the CVA is less than 44° [31], 48–50° [32], or 48.7° [33], the posture is defined as an unsuitable FHP.

In this study, the good posture was defined as a CVA higher than 48.7°, and a fair one had a range of 43.8–48.7°; a CVA less than 43.8° was considered a bad posture [6]. The results showed that the CVA of the low-FRR group (41.7°) was lower than that of the normal-FRR group (45.6°) by about 4°. This indicated that even if participants did not have any neck pain or symptoms, an awkward head posture, as measured by the FHP, was frequently observed during the VDT task in both the normal- and low-FRR groups.

The proportion of those with good posture for the moving monitor (32%) was higher than that for the fixed monitor (23%). The difference in the CVA ratios of all types of postures (good, fair, and bad) between the monitors was less than 5% in the normal-FRR group, whereas the low-FRR group showed a relatively large difference in the ratio, depending on the monitor type. For the low-FRR group, the ratio of bad postures decreased from 71.2% (fixed monitor) to 56.6% (moving monitor). Moreover, the ratio of those with good posture also increased from 8% (fixed monitor) to 21% (moving monitor).

Therefore, these results could be interpreted as showing that the moving monitor, which can change the height of the monitor periodically, helps prevent FHP. The effect of the moving monitor on posture correction was more remarkable in the low-FRR group than in the normal-FRR group.

### 4.2. Flexion Relaxation Ratio (FRR)

The FRP is an electrical silence response of the CES muscle during a full trunk flexion motion [12] and has also been found in neck muscles [16]. FRP has frequently been used to evaluate neck pains [31], and the FRR is one of the most frequently used variables in FRP studies.

Previous studies have shown that cervical FRR could be used to discriminate between neck-pain patients and healthy control groups because a relatively lower value of FRR has been found in neck-pain patients than in a healthy control group [18,19]. Murphy et al. (2010) reported that the FRR value in neck-pain patients (2.20) was significantly lower than in controls (4.24) [18]. Similarly, Maroufi et al. (2013) also indicated that the mean FRR of the neck-pain group (2.22) was significantly lower than that of the control group (4.88) [19]. The presence or absence of an FRP is usually defined by an FRR threshold value of 2.5 [27]. Thus, participants with FRR values higher than 2.5 were placed in the normal-FRR group, and the rest were assigned to the low-FRR group. The mean FRRs were 2.0 and 3.9 for the low-FRR and normal-FRR groups, respectively.

FRR has also been used as an indicator for quantifying the fatigue of CES and has become a sensitive marker for measuring fatigue associated with even mild discomfort [34,35]. In this study, a document task was carried out for 50 min to compare the FRR values, which decreased from 3.11 before the document writing task to 2.79 after it (Section 3.3); this means that the task of 50 min caused fatigue in the CES. This result was similar to those of previous studies [15,36]. Yoo et al. (2011) also reported that FRR could be used to evaluate the potential risk of neck discomfort during computer work [37].

In the healthy controls, FRR decreases after fatigue [15,20,36]; the greater activation of neck muscle could explain this during the relaxation phase (phase 3). After fatigue, the excitation-contraction coupling of muscle fiber is reduced [38] and increases the motor-unit firing rate to maintain similar force exertions, especially during isometric exertions [39]. Therefore, after fatigue, muscle activation in the relaxation phase (phase 3) increases to maintain the head load compared to the pre-fatigue trials (decreases the FRR value after the fatigue). In this study, the FRR values for both fixed and moving monitors were compared to evaluate the effect of the moving monitor. Although not statistically significant, there were some differences in the fatigue-induced FRR change according to the neck health condition. For the normal-FRR group, FRR decreased after the document task for both the moving monitor and the fixed monitor; this was in line with the results of several previous studies [15,20,34,40]. FRR decreased by about 0.262 after the document task when using the moving monitor, whereas the fixed monitor showed a relatively more significant decrease (0.494) than the moving monitor. It was noted that the fatigue in CES using the moving monitor was less than that of using the fixed monitor in the normal-FRR group.

One interesting result of the interaction effect of monitor type and pre/post on FRR in the low-FRR group (Figure 8) was that the FRR decreased after the task for both fixed and moving monitors; however, the FRR decrease for the moving monitor (0.374) after the document task was higher than that for the fixed monitor (0.147). Zabihhosseinian’s (2015) findings of FRR for pain and control groups showed that FRR decreased after fatigue in healthy controls and increased after fatigue in the neck-pain group [21]. It is essential to see that there is a potential difference in CES fatigue between healthy and neck-pain groups. This finding is in line with the result of our study.

This study has some limitations. First, only male adults participated in this study. Some studies have reported a higher prevalence of neck pain among females than males [41,42,43,44]. Therefore, the difference between genders should be analyzed in further studies. Second, the low-FRR group was smaller than the normal-FRR group. To overcome this limitation, an equal number of participants should be considered in future studies. Lastly, we tried to interpret clinical symptoms based on electrophysiology in this study; however, there is a limit to interpreting everything with just the muscle activity signals.

## 5. Conclusions

To evaluate the effect of the moving monitor, three types of variables (FRR, CVA, and CVA ratio) were used to quantify the fatigue and neck posture during the document task.

As the first research objective of this study, CVA and FRR were applied to evaluate neck posture and fatigue level quantitatively. As a result of testing 50 min document writing task, the neck posture was assessed according to the CVA value. Besides, the fatigue of the neck muscle was evaluated through the decrease of FRR value.

As the second research objective, using CVA and FRR, the different levels of fatigue and neck posture were evaluated according to the monitor type. The FRR results showed that both normal-FRR and low-FRR groups showed less fatigue after using the moving monitor than the fixed monitor. The CVA analysis showed that the frequency of FHP for the moving monitor was lower than that for the fixed monitor. In addition, the posture-correction effect of the moving monitor was more remarkable in the low-FRR group. To summarize, the moving monitor might cause less CES fatigue and less FHP than the fixed monitor.

## Figures and Tables

**Table 1:** Participant’s demographic data (Mean ± Standard deviation).

Neck Condition	FRR	Age (Year)	Weight (kg)	Height (cm)	Sitting Height (cm)
Normal-FRR group	3.9 ± 1.4	26.4 ± 2.5	74.6 ± 7.7	174.2 ± 3.8	92.0 ± 3.4
Low-FRR group	2.0 ± 0.5	27.6 ± 3.1	81.4 ± 14.3	172.9 ± 2.9	90.0 ± 2.9

FRR = flexion relaxation ratio.

**Table 2:** The interaction effect of monitor type and neck health condition.

FRR Group	Fixed Monitor			Moving Monitor		
	Good	Fair	Bad	Good	Fair	Bad
Normal-FRR	38.4%	16.4%	45.2%	42.0%	16.5%	41.5%
Low-FRR	8.0%	20.9%	71.2%	21.0%	22.4%	56.6%

RR = flexion relaxation ratio.

## References

[1] Lee I.S., Kim S.Y. (2020). Correlation among the cervical kyphotic angle, pain, and disability level in patients with temporomandibular disorders. Phys. Ther. Korea.

[2] Yoo W.G., Yi C.H., Kim M.H. (2006). Effects of a proximity-sensing feedback chair on head, shoulder, and trunk postures when working at a visual display terminal. J. Occup. Rehabil..

[3] Sauter S.L., Schleifer L.M., Knutson S.J. (1991). Work posture, workstation design, and musculoskeletal discomfort in a VDT data entry task. Hum. Factors.

[4] Mekhora K., Liston C.B., Nanthavanij S., Cole J.H. (2000). The effect of ergonomic intervention on discomfort in computer users with tension neck syndrome. Int. J. Ind. Ergon..

[5] Grace E.G., Sarlani E., Read B. (2002). The use of an oral exercise device in the treatment of muscular TMD. Cranio.

[6] Kim E.J. (2018). Effects of Turtle Neck Syndrome on Respiratory Volume According to Posture. Ph.D. Thesis.

[7] Kim S.W., Kim S.J., Son S.K., Dong S.O., Lee J.C., Shin D.J. (2013). Correlation between the head forward posture and the site of herniation of single level cervical intervertebral disc. J. Korea CHUNA Man. Med. Spine Nerves.

[8] Kim S.Y., Kim N.S., Jung J.H., Jo M.R. (2013). Effect of Forward Head Posture on Respiratory Function in Young Adults. J. Korean Phys. Ther..

[9] Cho J., Lee E., Lee S. (2017). Upper thoracic spine mobilization and mobility exercise versus upper cervical spine mobilization and stabilization exercise in individuals with forward head posture: A randomized clinical trial. BMC Musculoskelet. Disord..

[10] Yip C.H.T., Chiu T.T.W., Poon A.T.K. (2008). The relationship between head posture and severity and disability of patients with neck pain. Man. Therap..

[11] Quek J., Pua Y.H., Clark R.A., Bryant A.L. (2013). Effects of thoracic kyphosis and forward head posture on cervical range of motion in older adults. Man. Therap..

[12] Floyd W.F., Silver P.H.S. (1951). Function of erectores spinae in flexion of the trunk. Lancet.

[13] Panjabi M.M. (1992). The stabilizing system of the spine. Part I. Function, dysfunction, adaptation, and enhancement. J. Spinal Disord. Tech..

[14] Colloca C.J., Hinrichs R.N. (2005). The biomechanical and clinical significance of the lumbar erector spinae flexion-relaxation phenomenon: A review of literature. J. Manip. Physiol. Ther..

[15] Nimbarte A.D., Zreiqat M.M., Chowdhury S.K. (2014). Cervical flexion-relaxation response to neck muscle fatigue in males and females. J. Electromyogr. Kinesiol..

[16] Meyer J.J., Berk R.J., Anderson A.V. (1993). Recruitment patterns in the cervical paraspinal muscles during cervical forward flexion: Evidence of cervical flexion-relaxation. Electromyogr. Clin. Neurophysiol..

[17] Hashemirad F., Talebian S., Hatef B., Kahlaee A.H. (2009). The relationship between flexibility and EMG activity pattern of the erector spinae muscles during trunk flexion-extension. J. Electromyogr. Kinesiol..

[18] Murphy B.A., Marshall P.W., Taylor H.H. (2010). The cervical flexion-relaxation ratio: Reproducibility and comparison between chronic neck pain patients and controls. Spine.

[19] Maroufi N., Ahmadi A., Khatir S.R.M. (2013). A comparative investigation of flexion relaxation phenomenon in healthy and chronic neck pain subjects. Eur. Spine J..

[20] Nimbarte A.D., Zreiqat M., Ning X. (2014). Impact of shoulder position and fatigue on the flexion-relaxation response in cervical spine. Clin. Biomech..

[21] Zabihhosseinian M., Holmes M.W., Ferguson B., Murphy B. (2015). Neck muscle fatigue alters the cervical flexion relaxation ratio in sub-clinical neck pain patients. Clin. Biomech..

[22] Lindstrom L.H., Magnusson R.I. Interpretation of Myoelectric Power Spectra: A Model and Its Applications. Proceedings of the IEEE.

[23] Murata A., Uetake A., Matsumoto S., Takasawa Y. (2003). Evaluation of shoulder muscular fatigue induced during VDT tasks. Int. J. Hum. Comput. Interact..

[24] Niu H., Li R., Liu G., Pu F., Li D., Fan Y. Using EMG to Evaluate Muscular Fatigue Induced during Video Display Terminal Keyboard Use Task. Proceedings of the 7th Asian-Pacific Conference on Medical and Biological Engineering.

[25] Chea Y.W. (2002). The measurement of forward head posture and pressure pain threshold in neck muscle. J. Korean Phys. Ther..

[26] Kang J.H., Park R.Y., Lee S.J., Kim J.Y., Yoon S.R., Jung K.I. (2012). The effect of the forward head posture on postural balance in long time computer based worker. Ann. Rehabil. Med..

[27] Pialasse J.P., Dubois J.D., Choquette M.H.P., Lafond D., Descarreaux M. (2009). Kinematic and electromyographic parameters of the cervical flexion–relaxation phenomenon: The effect of trunk positioning. Ann. Phys. Rehabil. Med..

[28] Lee K.J., Han H.Y., Cheon S.H., Park S.H., Yong M.S. (2015). The effect of forward head posture on muscle activity during neck protraction and retraction. J. Phys. Ther. Sci..

[29] Harman K., Hubley-Kozey C.L., Butler H. (2005). Effectiveness of an exercise program to improve forward head posture in normal adults: A randomized, controlled 10-week trial. J. Man. Manip. Ther..

[30] Im B., Kim Y., Chung Y., Hwang S. (2016). Effects of scapular stabilization exercise on neck posture and muscle activation in individuals with neck pain and forward head posture. J Phys. Ther. Sci..

[31] Gurudut P., Welling A., Chodankar A. (2020). Effect of self-care exercises in forward head posture on craniovertebral angle and craniocervical flexion endurance: A pilot study. Indian J. Phys. Ther. Res..

[32] Watson D.H., Trott P.H. (1993). Cervical headache: An investigation of natural head posture and upper cervical flexor muscle performance. Cephalalgia.

[33] Salahzadeh Z., Maroufi N., Ahmadi A., Behtash H., Razmjoo A., Gohari M., Parnianpour M. (2014). Assessment of forward head posture in females: Observational and photogrammetry methods. J. Back. Musculoskelet. Rehabil..

[34] Shin S.J., An D.H., Oh J.S., Yoo W.G. (2012). Changes in pressure pain in the upper trapezius muscle, cervical range of motion, and the cervical flexion-relaxation ratio after overhead work. Ind. Health.

[35] DeVocht J.W., Gudavalli K., Gudavalli M., Xia T. (2016). Novel electromyographic protocols using axial rotation and cervical flexion-relaxation for the assessment of subjects with neck pain: A feasibility study. J. Chiropr. Med..

[36] Shin S.J., Yoo W.G. (2014). Changes in cervical range of motion, flexion-relaxation ratio and pain with visual display terminal work. Work.

[37] Yoo W.G., Park S.Y., Lee M.R. (2011). Relationship between active cervical range of motion and flexion–relaxation ratio in asymptomatic computer workers. J. Physiol. Anthropol..

[38] Allen D.G. (2004). Skeletal muscle function: Role of ionic changes in fatigue, damage and disease. Clin. Exp. Pharmacol. Physiol..

[39] Vukova T., Vydevska-Chichova M., Radicheva N. (2008). Fatigue-induced changes in muscle fiber action potentials estimated by wavelet analysis. J. Electromyogr. Kinesiol..

[40] Yoo W.G., Yoo I.G. (2014). Changes in the cervical FRR, shoulder muscle pain and position after continuous detailed assembly work. Work.

[41] Cagnie B., Danneels L., van Tiggelen D., de Loose V., Cambier D. (2007). Individual and work related risk factors for neck pain among office workers: A cross sectional study. Eur. Spine. J..

[42] Fernandes R.D.C.P., Carvalho F.M., Assunção A.Á. (2011). Prevalence of musculoskeletal disorders among plastics industry workers. Cad. Saude Publica.

[43] Skov T., Borg V., Orhede E. (1996). Psychosocial and physical risk factors for musculoskeletal disorders of the neck, shoulders, and lower back in salespeople. Occup. Environ. Med..

[44] Wang H., Naghavi M., Allen C., Barber R.M., Bhutta Z.A., Carter A., Casey D.C., Charlson F.J., Chen A.Z., Coates M.M. (2016). GBD 2015 Mortality and Causes of Death Collaborators. Global, regional, and national life expectancy, all-cause mortality, and cause-specific mortality for 249 causes of death, 1980–2015: A systematic analysis for the Global Burden of Disease Study 2015. Lancet.

